# Efficacy of two different self-expanding nitinol stents for atherosclerotic femoropopliteal arterial disease (SENS-FP trial): study protocol for a randomized controlled trial

**DOI:** 10.1186/1745-6215-15-355

**Published:** 2014-09-10

**Authors:** Sang Ho Park, Seung Woon Rha, Cheol Ung Choi, Eung Ju Kim, Dong Joo Oh, Yun Hyeong Cho, Woong Gil Choi, Seung Jin Lee, Yong Hoon Kim, Seung Hyuk Choi, Won Ho Kim, Ki Chang Kim, Jang Hyun Cho, Joo Han Kim, Sang Min Kim, Jang Ho Bae, Jung Min Bong, Won Yu Kang, Ju Yeol Baek, Jae Bin Seo, Woo Young Chung, Mahn Won Park, Sung Ho Her, Jon Suh, Min Woong Kim, Yeo Joo Kim, Hwan Jun Choi, Jae Wan Soh, SENS-FP Investigators

**Affiliations:** Department of Cardiology, Soonchunhyan, University Cheonan Hospital, 31 Soonchunhyang-6Gil, Dongnam-Gu, Cheonan, 330-721 Republic of Korea; Cardiovascular Center, Korea University Guro Hospital, 80 Guro-dong, Guro-gu, Seoul, 152-703 Republic of Korea; Cardiovascular Center, Myongji Hospital, 55, Hwasu-ro 14beon-gil, Deogyang-gu, Goyang, 412-826 Republic of Korea; Department of Cardiology, Konkuk University Chungju Hospital, 620-5 Kyohyun2-dong, Chungju, 380-704 Republic of Korea; Divison of Cardiology, Department of Internal Medicine, Kangwon National University School of Medicine, Baengnyeong-ro 156, Chuncheon, 200-722 Republic of Korea; Division of Cardiology, Department of Medicine, Samsung Medical Center, Sungkyunkwan University School of Medicine, 81 Irwon-Ro Gangnam-gu, Seoul, 135-710 Republic of Korea; Cardiovascular Center, Eulji Univesity Deajeon Hospital, Dunsan-dong 1306, Seo-gu, Deajeon, 302-799 Republic of Korea; Cardiovascular Center, Incheon Sa-Rang General Hospital, 726, Michuhol-daero, Nam-gu, Incheon, 402-835 Republic of Korea; Cardiovascular Center, St. Carollo General Hospital, 221, Sungwang-ro, Soonchun, 540-719 Republic of Korea; Cardiovascular Center, Chonnam University Hospital, 42 Jebong-Ro, Donggu, Gwangju, 501-757 Republic of Korea; Regional Cardiovascular Center, Division of Cariology, Department of Medicine, Chungbuk National University, 1473, Seobu-ro, Seowon-gu, Cheongju, 362-240 Republic of Korea; Cardiovascular Center, Konyang University Hospital, 158, Gwanjeodong-roSeo-gu, Daejeon, 302-812 Republic of Korea; Cardiovascular Center, Incheon, Hanlim General Hospital, 722, Jangje-ro, Gyeyang-gu, Incheon, 407-060 Republic of Korea; Cardiovascular Center, Gwangju Bohoon General Hospital, 99, Cheomdanwolbong-ro, Gwangsan-gu, Gwangju, 506-705 Republic of Korea; Cardiovascular Center, Cheongju St. Mary’s Hospital, 173-19, Juseong-ro, Cheongwon-gu Chungju, 173-19 Republic of Korea; Cardiovascular Center, Seoul National University Boramae Medical Center, 20, Boramae-ro 5-gil, Dongjak-gu, Seoul, 156-707 Republic of Korea; Department of Cardiology, Deajeon St. Mary’s Hospital, College of Medicine, The Catholic University of Korea, 64, Daeheung-roJung-gu, Deajeon, 301-723 Republic of Korea; Department of Cardiology, Cardiovascular center, Soonchunhyang University Bucheon Hospital, 170, Jomaru-ro, Wonmi-gu, Bucheon, 420-767 Republic of Korea; Department of Cardiology, Hanyang University Medical Center, Hanmaeum Hospital, 43-3, Sangnam-dong, Seongsan-gu, Changwon, 642-832 Republic of Korea; Department of Endocrinology & Metabolism, Soonchunhyang University Cheonan Hospital, 31 Soonchunhyang-6Gil, Dongnam-Gu, Cheonan, 330-721 Republic of Korea; Department of Plastic & Reconstructive Surgery, Soonchunhyang University Cheonan Hospital, 31 Soonchunhyang-6Gil, Dongnam-Gu, Cheonan, 330-721 Republic of Korea; Department of Orthopedic Surgery, Soonchunhyang University Cheonan Hospital, 31 Soonchunhyang-6Gil, Dongnam-Gu, Cheonan, 330-721 Republic of Korea

## Abstract

**Background:**

There have been few randomized control trials comparing the incidence of stent fracture and primary patency among different self-expanding nitinol stents to date. The SMART™ CONTROL stent (Cordis Corp, Miami Lakes, Florida, United States) has a peak-to-valley bridge and inline interconnection, whereas the COMPLETE™-SE stent (Medtronic Vascular, Santa Rosa, California, United States) crowns have been configured to minimize crown-to-crown interaction, increasing the stent's flexibility without compromising radial strength. Further, the 2011 ESC (European society of cardiology) guidelines recommend that dual antiplatelet therapy with aspirin and a thienopyridine such as clopidogrel should be administered for at least one month after infrainguinal bare metal stent implantation. Cilostazol has been reported to reduce intimal hyperplasia and subsequent repeat revascularization. To date, there has been no randomized study comparing the safety and efficacy of two different antiplatelet regimens, clopidogrel and cilostazol, following successful femoropopliteal stenting.

**Methods/Design:**

The primary purpose of our study is to examine the incidence of stent fracture and primary patency between two different major representative self-expanding nitinol stents (SMART™ CONTROL versus COMPLETE™-SE) in stenotic or occlusive femoropopliteal arterial lesion. The secondary purpose is to examine whether there is any difference in efficacy and safety between aspirin plus clopidogrel versus aspirin plus cilostazol for one month following stent implantation in femoropopliteal lesions. This is a prospective, randomized, multicenter trial to assess the efficacy of the COMPLETE™-SE versus SMART™ CONTROL stent for provisional stenting after balloon angioplasty in femoropopliteal arterial lesions. The study design is a 2x2 randomization design and a total of 346 patients will be enrolled. The primary endpoint of this study is the rate of binary restenosis in the treated segment at 12 months after intervention as determined by catheter angiography or duplex ultrasound.

**Discussion:**

This trial will provide powerful insight into whether the design of the COMPLETE™-SE stent is more fracture-resistant or effective in preventing restenosis compared with the SMART™ CONTROL stent. Also, it will determine the efficacy and safety of aspirin plus clopidogrel versus aspirin plus cilostazol in patients undergoing stent implantation in femoropopliteal lesions.

**Trial registration:**

Registered on 2 April 2012 with the National Institutes of Health Clinical Trials Registry (ClinicalTrials.gov identifier# NCT01570803).

## Background

Percutaneous transluminal balloon angioplasty for atherosclerotic femoropopliteal lesions has a high primary success rate of 90% without complications. However, restenosis rates are 60% within a year and restenosis rates increase to 70% for interventions on the superficial femoral artery where the lesion length is over 10 cm. Because of this, surgical treatment for restenosis was recommended by the Inter-Society Consensus for the Management of Peripheral Arterial Disease (TASC) in 2000 [1]. Later, a stainless steel stent was developed for atherosclerotic femoropopliteal lesions and adopted for clinical use. However, five randomized controlled trials failed to demonstrate any benefit of a stainless steel stent over balloon angioplasty alone [2–6].

The development of a nitinol stent has resulted in an innovative change in the interventional approach. Nitinol stents have contributed greatly to the lowering of restenosis rates and have also been shown to be superior to balloon angioplasty alone [7–10]. Based on these findings, the TASC II practice guidelines were developed [11]. Due to the development of the nitinol stent, restenosis rates have been reduced following femoropopliteal stenting. However, the use of a nitinol stent is only recommended when the result of a balloon angioplasty is not satisfactory as the ‘provisional concept’, and restenosis rates are still high in long lesions, such as TASC type C and D lesions.

The superficial femoral artery is the longest artery in human body and is located between two flexion points, the femoral joint and knee joint. Several types of forces are regularly applied to this vessel, such as flexion, longitudinal or lateral compression, torsion and others. Because of such features of the femoropopliteal artery, stent fracture can occur and several studies reported that the stent fracture can be a clinically significant factor in restenosis [12, 13].

Other reported risk factors for in-stent restenosis include stent length and the number of implanted stents (overlapping stenting), stent design, severity of calcification of the vessel, and stent elongation that may occur during deployment [14, 15]. Recently developed stent designs focus on reducing the rate of stent fracture to prevent restenosis. In the FESTO study [12], it was hypothesized that differences in nitinol stent designs affect stent fracture and restenosis rates. In this study, the Luminexx stent showed the highest stent fracture rate while the SMART™ stent showed the lowest stent fracture rate, with the fracture rate increasing with lesion length. Additionally, it was concluded that stent fracture is closely related to in-stent restenosis or reocclusion. Based on an *in vitro* study, Stefan Müller-Hülsbeck *et al*. claimed that there are differences in the performance of seven different types of stents in a mechanical fatigue test designed to predict stent fracture and, based on this, they also claimed that differences in stent design may play an important role in development of stent fracture [16].

Currently, several manufacturers produce nitinol stents for atherosclerotic femoropopliteal lesions, and all of them may differ in incidence of stent structure. As mentioned earlier, there are several laboratory, retrospective, or registered clinical studies on restenosis that compare the effect of bending, compression, and torsion on stent fracture rates in a variety of stent designs or types. However, well-designed, prospective, randomized, controlled clinical trials that compare restenosis rates among different stent types have rarely been conducted. Until now, the SUPER-SL study [14] is the only literature available for a randomized comparative study examining differences in primary patency rates between the SMART™ stent and Luminexx stent in long superficial femoral artery lesions (lesion length 5 to 22 cm). There is no randomized comparative study that compares two different types of nitinol stents in terms of stent fracture and primary patency rates in an Asian population, especially among new-generation nitinol stents.

The SMART™ CONTROL stent (Cordis Corp, Miami Lakes, Florida, United States) and the COMPLETE™-SE stent (Medtronic Vascular, Santa Rosa, California, United States) are both open-cell slotted tube designs, but they differ in connection shape between cell and cell or between crown and crown, and number of bridges. Because of such differences in design, Medtronic claim that the COMPLETE™-SE stent has exceptional elasticity and flexibility compared to other nitinol stents, without decreasing radial force.

Further, according to the 2011 ESC (European society of cardiology) guidelines [17], dual antiplatelet therapy with aspirin and clopidogrel is recommended at least for one month after the implantation of a bare metal stent into an atherosclerotic femoropopliteal lesion. Clinical research on the effectiveness of cilostazol in atherosclerotic peripheral arterial disease have been frequently reported. Recently, meta-analysis results have also reported that cilostazol is effective in reducing intermittent claudication [18]. However, while there are small-scale [19] or retrospective studies [20] that include a partial comparison showing a reduction in restenosis or revascularization rates in patients stented for femoropopliteal artery disease, there are few studies involving a large number of patients. Additionally, there are no randomized studies comparing the long-term effects of clopidogrel and cilostazol on reducing restenosis or revascularization rates in patients following femoropopliteal artery self-expanding nitinol stenting.

In this study, we will compare the incidence of stent fracture and primary patency between SMART™ CONTROL and COMPLETE™-SE stents. In addition, we would like to ascertain whether there are differences in efficacy and safety between aspirin plus clopidogrel versus aspirin plus cilostazol in patients undergoing stent implantation in femoropopliteal lesions.

## Method/Design

### Study objectives, hypothesis and design

**Figure 1:**
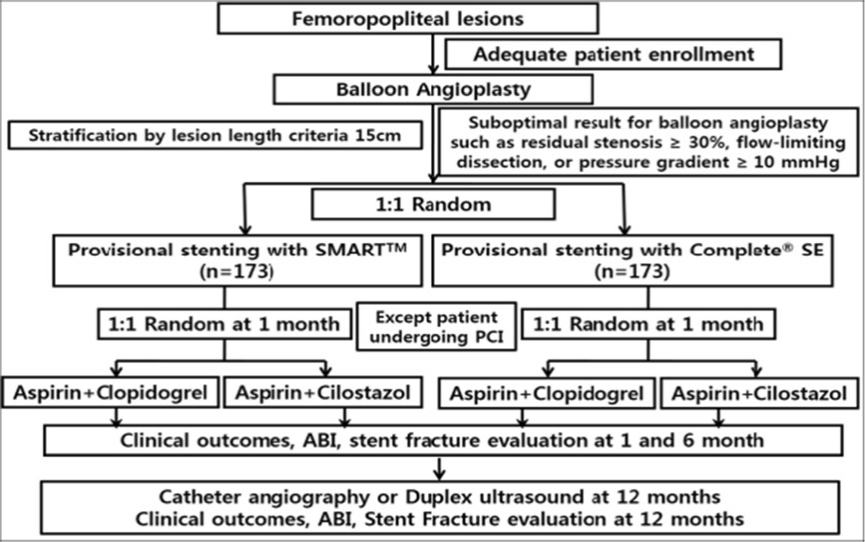
**Flow chart of the enrolled patients.** ABI: Ankle-brachial index; PCI: Percutaneous coronary intervention.

The primary purpose of our study is to examine and compare the incidence of stent fracture and primary patency between two different nitinol stents (SMART™ CONTROL versus COMPLETE™-SE) in stenotic or occlusive femoropopliteal arterial lesions. The secondary purpose is to ascertain whether there is difference in efficacy and safety between aspirin plus clopidogrel versus aspirin plus cilostazol in patients undergoing stent implantation in femoropopliteal lesions. This is a prospective, randomized, multicenter trial to assess the efficacy of the COMPLETE™-SE versus SMART™ CONTROL stent by provisional stenting after balloon angioplasty for stenotic or occlusive femoropopliteal arterial lesions. The protocol of the trial has been registered with the National Institutes of Health Clinical Trials Registry (registration number: NCT01570803) and a brief flow chart of the whole study is summarized in Figure 1.

### Study population

Patients at least 20 years of age who have moderate or severe intermittent claudication or critical limb ischemia (Rutherford score of 2 to 6, except any patient who has undergone or plans to take major amputation) will be screened for study enrollment. A patient will be enrolled if they meet all of the inclusion and have none of the exclusion criteria. Inclusion criteria consist of clinical and anatomical criteria. The clinical criteria includes symptomatic peripheral-artery disease with moderate to severe claudication (Rutherford score of 2 to 3), chronic critical limb ischemia with resting ischemic pain (Rutherford score of 4), or chronic critical limb ischemia with ischemic ulcers (Rutherford score of 5 to 6), and patient must provide written informed consent. The anatomical criteria includes stenosis of more than 50% of, or occlusion of the ipsilateral femoropopliteal artery, patent (≤50% stenosis) ipsilateral iliac artery, or concomitantly treatable ipsilateral iliac lesions (≤30% residual stenosis), and at least one patent (less than 50% stenosis) tibioperoneal run-off vessel. Patients meeting any of the following criteria will be excluded from the study: failure to provide written informed consent; a history of major bleeding within prior two months; known hypersensitivity or contraindication to any of the following medications: heparin, aspirin, clopidogrel, cilostazol, or contrast agent; acute limb ischemia; previous bypass surgery or stenting of the ipsilateral femoropopliteal artery; untreated inflow disease of the ipsilateral pelvic arteries (>50% stenosis or occlusion); patients who have undergone major amputation (amputation of above the ankle) or major amputation is planned or required; patients with a life expectancy of less than one year due to comorbidities.

### Randomization and interventions

Prior to the intervention, aspirin and clopidogrel will be administered at least 12 hours before the procedure. Prior to the intervention, 70 to 100 units/kg of unfractionated heparin will be administered. Endovascular interventions will be carried out percutaneously, by placing a 6–8-Fr sheath at the femoral artery via an antegrade approach or a contralateral crossover technique. In selected cases, a retrograde approach (from the distal superficial femoral artery, popliteal artery, or pedal arteries) and/or brachial approach will be allowed.

Diagnostic angiography will be performed in two different views at least 30 to 45° apart to evaluate the structure of the lesion. Femoropopliteal and tibial arteries will be visually checked for the presence of distal lesions. To document the precise location of the lesion and the site of the intervention, we will recommend the use of a ruler. In cases of total occlusion, both intraluminal and subintimal methods of recanalization will be allowed. In procedure, the use of other special devices will be allowed; for example reentry device OUTBACK™ catheter (Cordis Corp, Miami Lakes, Florida, United States) and Offroad catheter (Boston Scientific, Natick, MA, United states), CTO devices Frontrunner (Cordis Corp, Miami Lakes, Florida, United States) and Truepath (Boston Scientific, Natick, MA, United states), and atherectomy devices such as SilverHawk™ and TurboHawk™ (Covidien, Plymouth, Minnesota). After the successful passage of the guidewire, predilation of the target lesion with an optimally sized balloon will be performed prior to stent implantation. Recommended, minimal balloon dilation time is 120 seconds. Then, if there is a residual pressure gradient of ≥10 mmHg, residual stenosis of ≥30%, or flow-limiting dissection, the balloon angioplasty results will be considered as suboptimal results. Subsequently, web-based randomization for stent selection will be performed. Patients will be stratified into two groups, less than 15 cm or more than 15 cm, according to lesion length. Random allocation of the patients will be performed via a web-based computerized program separately managed at the Cardiovascular Intervention Research Institute (CIRI), Korea University Guro Hospital, Seoul, Republic of Korea. Patients will be randomized in a 1:1 manner according to two different stents (SMART™ CONTROL versus COMPLETE™-SE). The stents will be implanted to extend 10 mm proximally and distally from the margins of the target lesion with luminal narrowing of ≥50%. Spot stenting or full lesion coverage will be decided at physician’s discretion. When multiple stents are required, the margins of the stents should be overlapped by at least 10 mm. Adjuvant post-dilation after the stenting will be performed strictly within the stented segment, with up to 10% oversizing of the post-dilation balloon.

All ipsilateral iliac arterial lesions should be treated with angioplasty and/or stenting concomitantly and residual stenosis should be less than 50%. Treatment of tibioperoneal lesions is recommended only in cases of critical limb ischemia. There should exist at least one patent (<50% stenosis) tibioperoneal run-off vessel with good antegrade flow.

A final angiography will be performed after the intervention in both groups, with the use of the same angles and magnifications used in the baseline angiograms. Simultaneously, reference vessel diameter (RVD), minimal luminal diameter (MLD), percent diameter stenosis, and acute gain should be measured. The reference vessel diameter will be obtained from averaging 5-mm segments proximal and distal to the lesion. Technical success will be defined as the successful access and deployment of the stent and less than 30% diameter residual stenosis after the revascularization.

For the post-procedural medication, aspirin 100 mg and clopidogrel 75 mg will be administered once daily for one month. Then, patients of each group will be randomized by web-based randomization to receive either clopidogrel 75 mg or cilostazol 100 mg once daily in addition to aspirin 100 mg for at least 11 months in a 1:1 manner.

After enrollment and index procedure, clinical follow-up will be planned at 1, 6, and 12 months to evaluate clinical outcomes and ankle-brachial index score. Also, all patients will be recommended to have a follow-up catheter angiography or duplex ultrasound at 12 months according to local clinical practice. The incidence of stent fracture and binary patency will be assessed by plain X-ray and quantitative femoral angiography. The investigators will be urged to follow up on patients, either by office visits or by telephone contacts, as necessary. Patient adherence to the study drug and side effects will be checked and monitored at every outpatient visit. The decision of drug discontinuation will be discussed and checked under the physician’s recommendation.

### Outcome measures

The primary endpoint of this study is the rate of binary restenosis (stenosis of at least 50% of the luminal diameter) or PSVR (peak systolic velocity ratio) ≥2.5 or 0 (PSVR = peak systolic velocity within the area of stenosis divided by peak systolic velocity in a normal adjacent proximal artery segment) in the treated segment at 12 months after the intervention as determined by catheter angiography or duplex ultrasound. The secondary endpoints are as follows: 1) composite of binary restenosis and major stent fracture (moderate or severe stent fracture); 2) stent fracture rate according to fracture grade (minor, moderate, severe); 3) limb salvage rate free of above-the-ankle amputation; 4) sustained clinical improvement rate at 12-month follow-up; 5) repeated target lesion revascularization (TLR) rate; 6) repeated target extremity revascularization (TER) rate; 7) total reocclusion rate; 8) comparison of angiograph variables consisting of late loss and restenosis rate; 9) ankle-brachial index (ABI) at 12 months; 10) the rate of major adverse cardiovascular events (MACE) composed of all-cause death, myocardial infarction, repeat percutaneous coronary intervention (PCI), and stroke at 12 months; 11) incidence of geographic miss during stent deployment due to jumping and/or elongation; 12) rate of binary restenosis or PSVR ≥2.5 or 0 according to clopidogrel and cilostazol; and 13) major bleeding rate between clopidogrel and cilostazol group. The detailed definitions are summarized in the Appendix.

### Statistical analysis

#### Sample size calculation

From previous studies, including ABSOLUTE [7], FAST [8], and ASTRON [9], recurrence rates in femoropopliteal lesions after one year were 32%, 34.4%, and 37%, respectively - approximately 35% on average. The recurrence rate for the SMART™ CONTROL stent was 34.8% in the SUPER-SL study - a multicenter, randomized controlled trial that compared two self-expanding nitinol stents, the SMART™ CONTROL and Luminexx stents, in femoropopliteal lesions [14]. So far, there are limited data on the one-year primary patency rate of the COMPLETE™-SE stent. Based on previous research results, the SMART™ CONTROL stent group will be the control group, and the COMPLETE™-SE stent group will be the experimental group. Based on the SUPER-SL results, the one-year primary patency of the SMART™ CONTROL stent is estimated to be 65% and the one-year primary patency of the COMPLETE™-SE stent is estimated to be 80%, which is higher than that of the SMART™ CONTROL stent. Recurrence will be defined as more than 50% restenosis or total occlusion under invasive angiography, or if invasive angiography is not performed, PSVR ≥2.5 when evaluated with duplex ultrasound. The statistical significance level is set at 5%, the power of test is set as 80%, and the randomization ratio is set as 1:1. Test for proportion and chi-square method are used. Using standard sample size formula, it was calculated that 138 patients per group are needed for a total of 346 patients, after accounting for a 20% dropout rate.

### Statistical analysis

Categorical variables will be expressed as delivery rate when comparing baseline features between the SMART™ CONTROL and COMPLETE™-SE stent groups or the clopidogrel and cilostazol groups, and for categorical variables, comparisons between groups will use the chi-square or Fisher’s exact test. Continuous variables will be expressed as mean ± standard deviation, and for continuous variables, comparisons between groups will use the Student’s t-test. The primary and secondary endpoints will be analyzed using both intention-to-treat analysis (all subjects assigned to a treatment group) and per-protocol analysis (only subjects completed the treatment protocol). Study endpoints will also be analyzed per patient. For the primary endpoint (12-month binary restenosis rate) comparisons of the two patient groups will use the chi-square or Fisher’s exact test. At the end of the 12 months, cumulative restenosis rates will be calculated. All secondary endpoint analysis will be performed using the Student’s t-test, chi-square test, or Fisher’s exact test. Also, clinical outcomes such as revascularization of the target vessel, death, major cardiovascular events including myocardial infarction and cerebral infarction, and limb salvage rates will be analyzed using Kaplan-Meier survival estimates and a comparison of the groups will be performed using the log-rank test. A *P* value <0.05 is considered to be statistically significant. In order to evaluate the risk factors for recurrence, defined as restenosis or total occlusion, univariate and multivariate logistic regression analysis will be performed. Present research has concluded that there will be a very low chance of missing data to cause bias, and therefore, missing data for the major endpoints will be excluded from the analysis data. For repeated measurements for the secondary endpoints, last observation carried forward (LOCF) can be applied. The SPSS 20.0 (SPSS Inc, Chicago, Illinois, United States) statistics program will be used for all analysis.

### Trial organization

#### Executive committee

The Executive Committee will be composed of the study chairperson and selected members among the investigators. This committee is responsible for overseeing the administrative progress of the study and will approve the final trial design and protocol issued to the Data and Safety Monitoring Board (DSMB) and the clinical sites. This committee will also be responsible for reviewing the final results, determining the methods of presentation and publication, and selection of secondary projects and publications by members of the Steering Committee. The Executive Committee also holds the right to modify or stop the study prematurely based on recommendations from the DSMB.

### Data Safety Monitoring Board (DSMB)

The frequency of the DSMB meetings will be determined prior to study commencement. Additionally, the DSMB may call a meeting at any time if there is reason to suspect that safety is an issue. The DSMB is responsible for making recommendations regarding any safety or compliance issues throughout the course of the study and may recommend to the Executive Committee to modify or stop the study. However, all final decisions regarding study modifications rest with the Executive Committee.

All cumulative safety data will be reported to the DSMB and reviewed on an ongoing basis throughout enrollment and follow-up periods to ensure patient safety. Every effort will be made to allow the DSMB to conduct an unbiased review of patient safety information. All DSMB reports will be made available to the appropriate agencies upon request but will otherwise remain strictly confidential.

Prior to the DSMB’s first review of the data, the DSMB charter will be drafted. The DSMB will develop a consensus understanding of all trial endpoints and definitions used in the event adjudication process. All DSMB reports will remain strictly confidential, but will be made available to the regulatory body upon request.

### Clinical Event Adjudication Committee (CEAC)

The Clinical Events Committee (CEAC) is comprised of interventional and non-interventional cardiologists who are not involved in the study. The CEAC is charged with the development of specific criteria used for the categorization of clinical events and clinical endpoints in the study which are based on protocol. At the onset of the trial, the CEAC will establish explicit rules outlining the minimum amount of data required and the algorithm followed in order to classify a clinical event. All members of the CEAC will be blinded to the primary results of the trial. The CEAC will meet regularly to review and adjudicate all clinical events. The CEAC will also review and rule on all deaths that occur throughout the trial.

### Data coordination and site management

Data coordination and site management services will be performed at the Cardiovascular Center of the Korea University Guro Hospital.

### Ethical approval

This study has been approved by the institutional review boards of investigator’s centers (Soonchunhyang University Cheonan Hospital, reference number SCHCA 2013-02-006; Korea University Guro Hospital, reference number MD12018; Kwandong University Myongji Hospital, reference number 13-030; Human Research Kunkuk University Chungju Hospital, reference number 2013-006; Kangwon National University Hospital, reference number 2013-01-004; Samsung Seoul Medical Center, reference number SMC 2013-02-075-001; Incheon Sa-Rang Hospital, reference number 2013-4; St Carollo Hospital, reference number SCH 2013-119; Chonnam University Hospital, reference number CNUH-2013-021; Chungbuk National University Hospital, reference number CBNUH 2013-02-001-001; Konyang University Hospital, reference number 13-05; IS Incheon Hanlim Hospital, reference number 2013-3; Gwangju Bohoon Hospital, reference number 2013-5-1; Cheongju St Mary's Hospital, reference number IRB-5A-1; Seoul National University Boramae Hospital, reference number 20130806/26-2013-70/082; Deajeon Catholic Medical Center, reference number DIRB-00110_1-006; Soonchunhyang University Bucheon Hospital, reference number 2013-11-003; Hanmaeum Medical Center, reference number MD2013-0006; Hallym University Chuncheon Sacred Heart Hospital, reference number 2014-64). The investigator will obtain their written approval before being allowed to conduct and participate in the study. The investigator is also responsible for fulfilling any conditions of approval imposed by the institutional review board, such as regular reporting, study timing, and so on. The investigator will provide the Sponsor with copies of such approvals and reports.

## Discussion

Endovascular revascularization is a minimally invasive therapy for the treatment of patients with femoropopliteal arterial disease who suffer from intermittent claudication or critical limb ischemia. Technology has rapidly evolved during the last decade and there is now a growing body of experience in the treatment of even complex cases [21]. However, the femoropopliteal artery remains a challenging area for endovascular treatment. During movements, various forces are exerted on this vessel. Therefore the use of intravascular stents in the femoropopliteal artery continues to be controversial due to the potential for compression and fracture. As mentioned previously, recent stent design improvements focus on decreasing stent fracture rates which can negatively impact patency rates by rearranging strut alignment.

### Rationale of randomized trials comparing two different generation stents

The factors associated with in-stent restenosis after stent implantation in femoropopliteal lesion have been known to be very various; patient characteristics, lesion characteristics, and so on. Recently, various self-expandable nitinol stents have been manufactured by many companies and they each claim that the design of their product is superior in terms of radial force, stent deformity or fracture, and deployment system. As aforementioned, several studies have shown that stent fracture might be associated with in-stent restenosis. Also geographic miss, due to stent elongation or jumping during stent deployment, may be associated with stent fracture and in-stent restenosis [22].

On the other hand, the design of self-expandable nitinol stents might be different depending on the time they were developed; first-generation nitinol stents (such as Luminexx™ and SMART™) showed a remarkably high rate of stent strut fracture [22]. A second generation of slotted tube nitinol stents has been developed. These stents have better flexibility by reducing the number of connections between cells or crowns and configuring the spiral orientation of these interconnections [23]. Several studies have reported that these nitinol stents are more fracture-resistant and more flexible, with some of them providing superior patency rate (such as Life™ and Everflex™) [22, 24].

**Figure 2:**
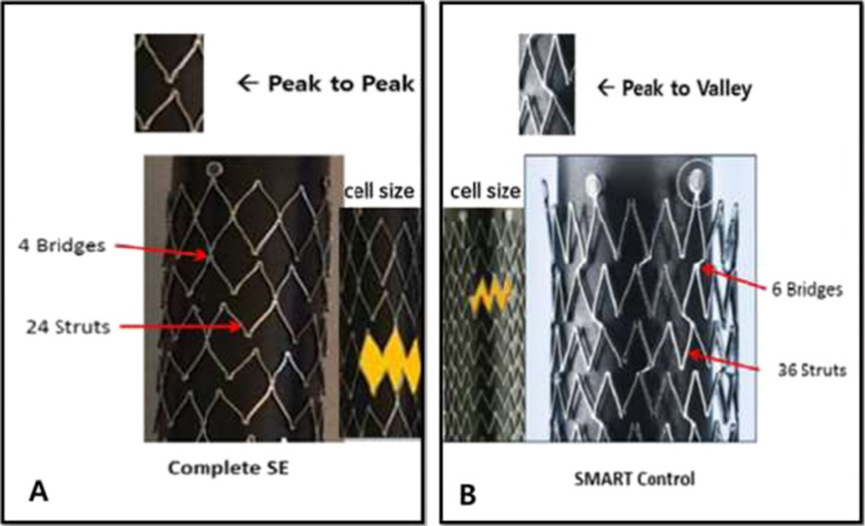
**Comparison for stent design. A**; Complete-SE stent design, **B**; SMART™ CONTROL stent design. Complete-SE stent is different to SMART™ CONTROL stent in that the configuration of interconnection of Complete-SE has peak-to-peak connection, fewer bridges (4 versus 6) and struts (24 versus 36), a larger cell size, and a more spiral orientation of interconnection, compared to the SMART™ CONTROL stent. On the other hand, the SMART™ CONTROL stent has the peak-to-valley bridge, more bridges, a smaller cell size, and inline interconnection.

However, an important limitation to their studies is that they were non-randomized studies of relative small sample size or were confined to *in vitro*. As of yet, a multicenter randomized controlled trial for direct comparison of stent fracture and primary patency between two different nitinol stents has not been done except for one study: SMART™ versus Luminexx stent (Super SL trial) [14]. SMART™ and Luminexx stents have been classified as first generation self-expandable nitinol stents. The Complete-SE stent (Figure 2A) of Medtronic company is different to the SMART™ CONTROL stent (Figure 2B) of Cordis company in that the configuration of interconnection of Complete-SE has peak-to-peak connection, fewer bridges (4 versus 6) and struts (24 versus 36), a larger cell size, and a more spiral orientation of interconnection, compared to the SMART™ CONTROL stent. On the other hand, the SMART™ CONTROL stent has the peak-to-valley bridge, more bridges, a smaller cell size, and inline interconnection. Medtronic have claimed Complete-SE has been configured to minimize cell-to-cell and bridge-to-bridge interaction, increasing the stent’s flexibility without compromising radial strength. We made the hypothesis that the design of the Complete-SE stent might be more fracture-resistant or effective for in-stent restenosis, compared with the SMART™ CONTROL stent.

### Rationale of randomized trials comparing clopidogrel and cilostazol following femoropopliteal stenting

To date, in many previous reports, dual antiplatelet therapy consisting of aspirin and clopidogrel has proven to decrease the incidence of cardiovascular death, myocardial infarction, and revascularization without an increase in major bleeding in patients who have undergone PCI regardless of stent type (bare metal stent or drug-eluting stent) [25–27]. In 2011 the ESC guidelines recommended dual antiplatelet therapy with aspirin and a thienopyridine such as clopidogrel for at least one month after infra-inguinal bare metal stent implantation (Class I, Level C). However, there has been no definite evidence or guideline for the optimal antiplatelet agent after stent implantation beyond one month later [17]. There have been many studies for the efficacy of thienopyridines in peripheral arterial disease. In the Swedish Ticlopidine Multicenter Study (STIMS) 687 patient of intermittent claudication were treated with ticlopidine or placebo, and the incidence of fatal or non-fatal myocardial infarction and stroke was reduced in the ticlopidine group [28]. A randomized blinded trial of clopidogrel versus aspirin in patients at risk of ischemic events (CAPRIE) demonstrated that clopidogrel was better in treating peripheral artery disease than aspirin (relative risk reduction in composite end point: 23.8%) [29]. Based on this trial, clopidogrel has been approved for prevention of ischemic events in patients with peripheral artery disease by the United State Food and Drug Administration [29]. Clopidogrel is effective in reducing cardiovascular events in a subgroup of patients with symptomatic peripheral arterial disease, with or without other clinical evidence of cardiovascular disease [11]. In the CHARISMA (Clopidogrel for High Atherothrombotic Risk and Ischemic Stabilization, Management, and Avoidance) trial, the large number of patients with documented prior myocardial infarction, ischemic stroke, or symptomatic peripheral artery disease appeared to derive significant benefit from dual antiplatelet therapy with clopidogrel plus aspirin beyond aspirin alone [30]. In the CASPAR (Results of the randomized, placebo-controlled clopidogrel and acetylsalicylic acid in bypass surgery for peripheral arterial disease) trial, the combination of clopidogrel plus aspirin did not improve limb or systemic outcomes in the overall population of peripheral artery disease patients requiring below-knee bypass grafting [31]. Subgroup analysis suggests that clopidogrel plus aspirin confers benefit in patients receiving prosthetic grafts (Hazard ratio: 0.65; 95% CI: 0.45-0.95; *P* = 0.025) without significantly increasing risk of major bleeding [31]. With the potential benefit of cilostazol on vascular function *in vitro*, there have been several previous efforts to prove the efficacy of cilostazol in patients undergoing endovascular therapy or stent implantation in peripheral arterial disease. For example, Soga *et al*. and Iida *et al.* have reported that cilostazol may improve amputation-free survival and limb salvage rate after endovascular therapy for infrainguinal disease in patients with critical limb ischemia and intermittent claudication [19, 32–34]. Also, they have demonstrated that cilostazol reduced restenosis after superficial femoral artery stenting with a self-expandable nitinol stent and it seems to be more effective in patients who are at a high risk of restenosis [35]. However, very few trials have not effectively or properly addressed the direct comparison for the efficacy and safety between clopidogrel and cilostazol. This trial is designed to evaluate the efficacy and safety between aspirin plus clopidogrel versus aspirin plus cilostazol in patients undergoing stent implantation in femoropopliteal lesions.

In conclusion, we still do not know whether there is a difference in primary patency and stent fracture between two self-expandable nitinol stents with a different design (SMART™ CONTROL versus COMPLETE™-SE) in stenotic or occlusive femoropopliteal arterial lesions. Furthermore, the efficacy and safety between aspirin plus clopidogrel versus aspirin plus cilostazol in patients undergoing stent implantation in femoropopliteal lesions will be evaluated. We hope to address these issues in the SENS-FP trial, whereby we will enroll a large unselected population of patients treated with stent implantation for significant peripheral arterial disease.

## Trial status

Recruitment is ongoing.

## Appendix

### Endpoints definitions

Binary restenosis: defined as a reduction in the luminal diameter of >50% according to the worst angiographic view at the narrowest site within the treated segment plus the 10-mm segments proximal and distal to the treated segment by catheter angiography or PSVR ≥2.5 (PSVR = peak systolic velocity within the area of stenosis divided by peak systolic velocity in a normal adjacent proximal artery segment) or total occlusion by duplex ultrasound. Stent fracture rate according fracture grade (minor, moderate, severe) [12]: plain X-ray examinations for the stent fracture are used or performed using at least two different angulations (30 to 45° difference) and the highest available magnification. Stent fracture is classified as minor (single strut fracture), moderate (fracture of more than 2 struts), or severe (complete separation of stent segments). Target lesion revascularization (TLR): defined as any repeat percutaneous intervention of the target lesion or bypass surgery of the target vessel performed for restenosis because of a return of ischemic symptoms, decrease of at least 1 Rutherford category, decrease in the ankle brachial index of >0.15, or restenosis of >50% as measured by catheter angiography. The target lesion is defined as the treated segment from 10 mm proximal to the stent and to 10 mm distal to the stent. Target extremity revascularization (TER): defined as any repeat percutaneous intervention of the target extremity or bypass surgery of the target extremity performed for restenosis because of a return of ischemic symptoms, decrease of at least 1 Rutherford category, decrease in the ankle brachial index of >0.15, or restenosis of >50% as measured by catheter angiography. Limb salvage: defined as survival free of amputation. Amputation is defined as above-ankle amputation of the index limb. Sustained clinical improvement rate [36, 37]: defined as persistent ABI value ≥0.15 and persistent improvement of ≥1 class according to Rutherford throughout follow-up when compared with baseline without the need for repeat TLR in surviving patients. Late loss: defined as a change in MLD from final angiogram to follow-up. Major adverse cardiac events (MACE): defined as composite of all-cause death, myocardial infarction, cerebrovascular stroke, and target lesion revascularization.

### Immediate outcomes

Acute lesion success: defined as a residual stenosis of ≤30% without flow-limiting dissection in the worst angiographic view. Definition of suboptimal result: residual pressure gradient of >10 mmHg, residual stenosis of >30%, or flow-limiting dissection. Acute hemodynamic success: defined as a ≥ 0.15 improvement in the ankle-brachial index from pre-procedure to immediately post-procedure (discharge). Clinical success: defined as an improvement of baseline symptoms by at least 1 Rutherford category that was sustained throughout follow-up with no additional intervention.

### QCA (Qualitative Comparative Analysis) data definition and variables

Reference vessel diameter (obtained from averaging 5-mm segments proximal and distal to the lesion), minimal luminal diameter (MLD), acute gain (change in MLD from baseline to post-intervention), late loss (change in MLD from the final angiogram to follow-up), percent diameter stenosis, total reocclusion rate, and binary (>50%) restenosis rate [38].

### Ankle-brachial index (ABI) calculation

Measurement of highest systolic pressure in both arms, measurement of systolic pressure in both legs, use highest ankle pressure (dorsalis pedis or posterior tibialis) for each leg, and calculate ratio of each ankle to brachial pressure by dividing each ankle by highest brachial pressure.

### Bleeding and hemorrhagic complications

An episode of bleeding is defined by the TIMI (Thrombolysis In Myocardial Infarction) criteria [39] as:

Major: overt clinical bleeding (or documented intracranial or retroperitoneal hemorrhage) associated with a drop in hemoglobin (Hgb) of greater than 5 g/dl (0.5 g/l) or in hematocrit (Hct) of greater than 15% (absolute). A patient who experiences an intracranial hemorrhage should be considered to have a major hemorrhage. Minor: overt clinical bleeding associated with a fall in hemoglobin of 3 to less than or equal to 5 g/dl (0.5 g/l) or in hematocrit of 9% to less than or equal to 15% (absolute). None: no bleeding event that meets the major or minor definition. In calculating the fall in hemoglobin or hematocrit, a transfusion of whole blood or packed red blood cells is counted as 1 g/dl (0.1 g/l) hemoglobin or 3% absolute in hematocrit. This would be in addition to the actual fall in hemoglobin or hematocrit. To account for transfusion, hemoglobin and hematocrit measurements will be adjusted for any packed red blood cells or whole blood given between baseline and post-transfusion measurements. A transfusion of one unit of blood will be assumed to result in an increase of 1 g/dL in Hgb or of 3% in Hct. Thus, to calculate the true change in Hgb or Hct if there has been an intervening transfusion between two blood measurements, the following calculations should be performed:

The following will be classified as ‘instrumented’ major bleeding that is considered to be associated with the catheterization laboratory visit: major percutaneous entry site: bleeding occurred at the percutaneous entry site during or after the catheterization laboratory visit until discharge. The bleeding should require a transfusion and/or prolong the health care facility stay, and/or cause a drop in Hgb >5 g/dL. Bleeding at the percutaneous entry site can be external or a hematoma >10 cm for femoral access or >2 cm for radial access or >5 cm for brachial access. major retroperitoneal, gastrointestinal, genital, and urinary: bleeding occurred during or after the catheterization laboratory visit until discharge. The bleeding either requires surgical intervention (for example, to relieve nerve compression), and/or requires a transfusion and/or prolongs the health care facility stay, and/or causes a drop in hemoglobin > 5.0 g/dL. major other or unknown: bleeding occurred at other or unknown locations during or after the catheterization laboratory visit until discharge. The bleeding should require a transfusion and/or prolong the healthcare facility stay and/or cause a drop in Hgb > 5 g/dL.

## Abbreviations

CTO: Chronic total occlusion
ECS: European Society of Cardiology
U.S. FDA: United State Food and Drug Administration
PAD: Peripheral arterial disease
PCI: Percutaneous coronary intervention
PSVR: Peak systolic velocity ratio
SFA: superficial femoral artery
TASC: Transatlantic Inter-Society Consensus.
